# Taxonomic studies on the ant genus *Ponera* Latreille, 1804 (Hymenoptera, Formicidae), with the description of a new species from India

**DOI:** 10.3897/zookeys.526.5971

**Published:** 2015-10-08

**Authors:** Himender Bharti, Joginder Singh Rilta

**Affiliations:** 1 Department of Zoology & Environmental Sciences, Punjabi University, Patiala, Punjab, 147002, India

**Keywords:** Ponerinae, new record, new species, north-eastern Himalaya

## Abstract

Four species of the ant genus *Ponera* Latreille, 1804, are recorded from India. The present study reports one new species *Ponera sikkimensis* **sp. n.**, a divergent population of *Ponera indica* Bharti & Wachkoo, 2012 and one new record, *Ponera paedericera* Zhou, 2001 from India. An identification key and distributions for the four known Indian species of *Ponera* based on the worker caste are provided.

## Introduction

The ant genus *Ponera* belongs in subfamily Ponerinae, and is currently represented by 56 extant and five fossil-based species (Bolton 2014). The genus was revised worldwide by Taylor (1967) and since then a number of additional species have been described by various workers (Terayama 1986, 1996, 2009; Perrault 1993; Xu 2001a, b; Zhou 2001; Csősz and Seifert 2003; Dlussky 2009; Bharti and Wachkoo 2012). The worker caste of *Ponera* superficially resembles those of some other Ponerinae genera (*Hypoponera*, *Cryptopone*, *Euponera*, and *Pseudoponera*) but can be distinguished from these due to the presence of an anterior fenestra in the subpetiolar process (a few species of *Hypoponera* apparently possess fenestrae, but lack paired posterior teeth on the subpetiolar process, which is a further character typifying *Ponera*). Additionally, *Ponera* has two maxillary palp segments, while *Hypoponera* has a one-segmented maxillary palp. Some other genera (*Belonopelta* and *Emeryopone*) also have an anterior fenestra in the subpetiolar process, but they have narrow mandibles with long attenuated teeth. *Ponera* differs from them on the basis of its typical triangular mandibles with only short teeth (Schmidt 2013; Schmidt and Shattuck 2014).

*Ponera* is currently represented by two species described from India: *Ponera indica* Bharti & Wachkoo, 2012 and *Ponera taylori* Bharti & Wachkoo, 2012. The present study reports one new species (*Ponera sikkimensis* sp. n.), a divergent population of *Ponera indica* Bharti & Wachkoo, 2012 and one new record (*Ponera paedericera* Zhou, 2001) from India. With the addition of these species, the genus is now represented by four species in India, which are distributed in Himalayan regions. A revised key is provided herewith. Two further species, *Ponera affinis* Jerdon, 1851 and *Ponera pumila* Jerdon, 1851 were described earlier from Malabar, India. Due to inadequate original descriptions and a lack of type material these have already been considered *incertae sedis* in the genus (Bharti 2008, 2011; Bharti and Wachkoo 2012).

## Materials and methods

The specimens were collected using the Winkler extractor method. Taxonomic analysis was conducted on a Nikon SMZ 1500 stereo zoom microscope with maximum magnification of 112.5×. For digital images, an MP (Micro Publisher) digital camera was used on the same microscope with Auto-Montage software (Syncroscopy, Division of Synoptics, Ltd.). Later, images were cleaned with Adobe Photoshop CS5 and Helicon Filter 5. Holotype of new species has been deposited in PUAC (Punjabi University Patiala Ant Collection at Department of Zoology and Environmental Sciences, Punjabi University, Patiala, Punjab, India). Measurements were recorded in millimeters on a Nikon SMZ 1500 stereo zoom microscope. The comparative morphometric data of the species are listed in Table 1. Morphological terminology for measurements and indices is as follows:

**Head Length (HL)** Maximum length of head in dorsal view, measured in as a straight line from the anterior most point of the median clypeal margin to the midpoint of the occipital margin.

**Head Width (HW)** Maximum width of head in dorsal view.

**Head Size (HS)** Head size, arithmetic mean of HL and HW.

**Scape Length (SL)** Maximum length of the scape excluding the basal neck and condyle.

**Pronotal Width (PrW)** Maximum width of pronotum in dorsal view.

**Weber’s Length (WL)** Mesosoma measured in lateral view from the anterior surface of the pronotum (excluding the collar) to the posterior margin of the propodeal lobes.

**Petiole Height (PH)** Maximum height of the petiole in profile from the apex of subpetiolar process to dorsal most point.

**Petiole Width (PW)** Maximum width of the petiole in dorsal view.

**Petiole Length (PL)** In profile, the distance from the midpoints of the curves where the anterior and posterior faces of the node meet the anterior and posterior peduncles.

**Cephalic Index (CI)** Cephalic index: HW × 100/HL.

**Scape Index (SI)** Scape index: SL × 100/HW.

**Petiole Node Index (PNI)** Petiolar node index: PW × 100/PrW.

**Lateral Petiole Index (LPI)** Lateral petiolar index: PL × 100/PH.

**Dorsal Petiole Index (DPI)** Dorsal petiole index: PW × 100/PL.

**Ocular Index (OI)** (sexuals only) Maximum diameter of eye divided by head width.

**Table 1.** Average worker measurements with standard deviation and minimum and maximum values in brackets.

Species	HL	HW	HS	SL	PrW	WL	PH	PW	PL	CI	SI	PNI	LPI	DPI
*Ponera indica* (type material) (n=12)	0.445±0.009 [0.430,0.460]	0.418±0.0058 [0.410,0.430]	0.432±0.006 [0.420,0.440]	0.315±0.015 [0.280,0.330]	0.302±0.0096 [0.280,0.310]	0.585±0.0018 [0.560,0.610]	0.289±0.008 [0.270,0.300]	0.229±0.001 [0.210,0.240]	0.104±0.009 [0.90,0.120]	94.4±2.24 [91.3,97.8]	74.1±5.2 [65.1,80.5]	74.5±5.12 [67.7,79.3]	36.7±21.37 [34.5,40]	221.4±21.44 [200,266.7]
*Ponera indica* (divergent population from North-east Himalaya) (n=13)	0.448±0.0016 [0.43,0.49]	0.396±0.013 [0.37,0.43]	0.421±0.014 [0.41,0.46]	0.289±0.016 [0.27,0.33]	0.248±0.016 [0.22,0.28]	0.55±0.06 [0.51,0.66]	0.246±0.015 [0.22,0.28]	0.216±0.029 [0.18,0.25]	0.166±0.016 [0.15,0.21]	88.35±1.653 [86.67,90.69]	73±3.068 [69.23,76.74]	87.21±9.709 [72,96]	68.11±5.906 [59.25,75]	130.69±19.755 [104.76,160]
*Ponera taylori* (n=12)	0.650±0.0219 [0.600,0.670]	0.585±0.019 [0.550,0.610]	0.620±0.0150 [0.595,0.640]	0.440±0.001 [0.430,0.460]	0.390±0.008 [0.380,0.400]	0.843±0.042 [0.740,0.890]	0.372±0.016 [0.350,0.390]	0.241±0.011 [0.200,0.260]	0.183±0.015 [0.160,0.200]	89.3±2.81 [83.3,93.8]	75.5±2.56 [71.7,81.8]	62.8±2.11 [60.5, 65]	47.8±2.99 [44.7,52.6]	113.2±9.02 [120,144.4]
*Ponera paedericera* (n=4)	0.735±0.020 [0.71,0.76]	0.602±0.015 [0.59,0.62]	0.667±0.015 [0.65,0.68]	0.485±0.019 [0.47,0.51]	0.41±0.033 [0.37,0.45]	0.802±0.022 [0.78,0.83]	0.38±0.033 [0.34,0.42]	0.395±0.005 [0.39,0.40]	0.267±0.012 [0.25,0.28]	81.98±1.710 [80.26,83.78]	80.55±4.400 [75.80,86.44]	96.75±6.818 [88.89,105.40]	70.85±7.934 [64.28,82.35]	147.89±6.66 [139.28,156]
*Ponera sikkimensis* sp.n. (n=3)	0.433±0.011 [0.42,0.44]	0.396±0.005 [0.39,0.40]	0.415±0.008 [0.40,0.42]	0.286±0.011 [0.28,0.30]	0.243±0.011 [0.23,0.25]	0.516±0.005 [0.51,0.52]	0.236±0.011 [0.23,0.25]	0.18±0 [0.18]	0.17±0 [0.17]	91.55±1.120 [90.91,92.85]	72.30±3.995 [70,76.92]	74.08±3.614 [72,78.26]	80.75±11.853 [73.91,94.44]	105.89±0 [105.89]

### Acronym of depository

PUAC “Punjabi University Patiala Ant Collection” at Department of Zoology and Environmental Sciences, Punjabi University, Patiala, Punjab, India.

## Results

### Ponera sikkimensis sp. n.

Taxon classificationAnimaliaHymenopteraFormicidae

http://zoobank.org/FB59D7A2-D2D6-4B82-A1F9-3F2904D7256D

Figs 1–3

#### Type locality.

India, Sikkim: Phadamchen, 27°12.75'N, 88°37.22'E, 1040 m, leaf litter, Winkler, 30 May 2012, Joginder Singh leg.

#### Type material.

Holotype worker and two paratype workers with same data as of holotype [PUAC].

#### Holotype measurements.

HL 0.42; HW 0.39; HS 0.40; SL 0.30; PrW 0.25; WL 0.52; PH 0.23; PL 0.17; PW 0.18; CI 92.85; SI 76.92; PNI 72; LPI 74; DPI 105.89.

*Head*: roughly oval in shape, distinctly longer than broad, sides convex, occipital margin concave, occipital corners rounded. Mandibles each with five well-developed teeth, Eyes small, composed of 3-4 indistinct facets. Anterior margin of clypeus concave. Apex of scape does not reach the midpoint of the occipital margin when laid straight back from its insertion in full-face view; funiculus incrassate toward apex; antennal club with four segments.

*Mesosoma and petiole*: In lateral view dorsum of mesosoma weakly convex, in dorsal view promesonotal suture distinct; metanotal groove indistinct. Dorsum of propodeum about as long as declivity, declivity flat, posterodorsal corner rounded. Petiole broader than long in dorsal view, dorsal face convex, in profile view, anterior and posterior faces straight, in dorsal view node roughly semicircular, anterior and lateral borders forming a single arc, posterior border weakly concave. Subpetiolar process with oval fenestra, anteroventral corner blunt, posteroventrally with enlarged teeth.

*Gaster*: Cinctus of second gastral tergite with cross ribs.

*Sculpture*: Head, mesosoma and gaster sparsely punctate, Petiolar dorsum more strongly punctate; propodeal declivity, posterior face of petiole and gastral apex smooth and shining. Mandibles shining with scattered punctures.

*Pilosity*: Dorsum of head, mesosoma, petiole and gaster with dense decument pubescence. Erect hairs present on anterior portion of head and posterior half of gaster.

*Colour*: Head brownish and dull; rest of body light brown and shining; mandibles, antennae and legs yellow.

**Figures 1–3.**
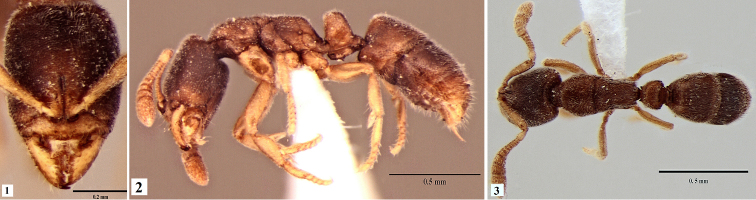
*Ponera sikkimensis* sp. n. worker **1** head in full-face view **2** body in profile view **3** body in dorsal view.

#### Etymology.

The species is named after the state of Sikkim.

#### Remarks.

*Ponera sikkimensis* sp. n. is somewhat similar to *Ponera longlina* Xu, 2001, described from China. It can be distinguished from the latter by the following combination of characters: head roughly oval in shape; mandible with five well-developed teeth, eyes with 3-4 indistinct facets; fenestra in subpetiolar process oval in shape; propodeum and petiole with stronger punctures; petiolar node relatively low and narrow. In *Ponera longlina* the head is roughly square in shape; mandibles each with three well-developed teeth at the apical margin followed by small denticles; eyes with single facet; fenestra in subpetiolar process circular in shape; propodeum and petiole smooth; petiolar node higher and relatively broad.

### Ponera indica

Taxon classificationAnimaliaHymenopteraFormicidae

Bharti & Wachkoo, 2012

#### Material examined.

India, Sikkim: Phadamchen, 27°12.75'N, 88°37.22'E, 1040 m, leaf litter, Winkler, 1 June 2012, 13 workers and 1 queen, Joginder Singh leg. Holotype worker with labels, “India, Himachal Pradesh, Terrace, 31.9234°N, 75.9294°E, 430 m, 12 October, 2008, Winkler”. Paratypes: 5 workers with same data as of Holotype, 1 worker and 1 gyne, India, Himachal Pradesh, Andretta, 32.0744°N, 76.5856°E, 940 m, 11 June, 2010, hand picking; 5 workers, India, Himachal Pradesh, Mandi, 31.7080°N 76.9318°E?, 800 m, 27 June, 2010, soil core (PUAC).

#### Remarks.

The *Ponera indica* material collected from Sikkim (north-eastern Himalaya) possibly represents a divergent population, as the species was originally described from north-western part of Himalaya. At present, the morphological differences outlined do not substantiate its status as a distinct species. The intraspecific variation includes: head rectangular in shape, occipital margin concave; mandibles each with three well- developed teeth followed by small denticles; fenestra in subpetiolar process circular; standing pilosity sparse; eyes composed of 1-2 indistinct facets; apex of scape does not reach the midpoint of posterior cephalic margin, CI 86.67–90.69; LPI 59.25–75; DPI 104.76–160. However, in the population representing type material of *Ponera indica*, the head is more oval in shape, the occipital margin straight; and the mandibles each with three well-developed teeth and without small denticles; the subpetiolar fenestra is oval in shape, and pilosity is abundant, eyes composed of 3-4 indistinct facets; apex of scape reaches the posterior cephalic margin, CI 91.3–97.8; LPI 34.5–40; DPI 200–266.7.

### Ponera paedericera

Taxon classificationAnimaliaHymenopteraFormicidae

Zhou, 2001

Figs 4–6

#### Material examined.

India, Arunachal Pradesh: Dirang, 27°21.50'N, 92°14.46'E, 1634m, 29 September 2013, Winkler, 4 workers and 1queen, Joginder Singh leg.

For complete description see Zhou (2001).

**Figures 4–6.**
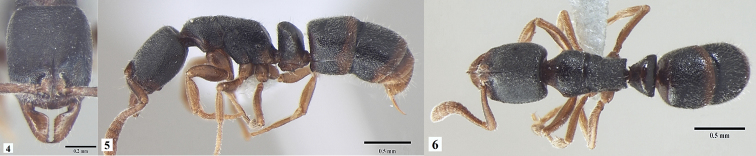
*Ponera paedericera* worker **4** head in full-face view **5** body in profile view **6** body in dorsal view.

#### Global distribution.

China, India.

**Figure 7.**
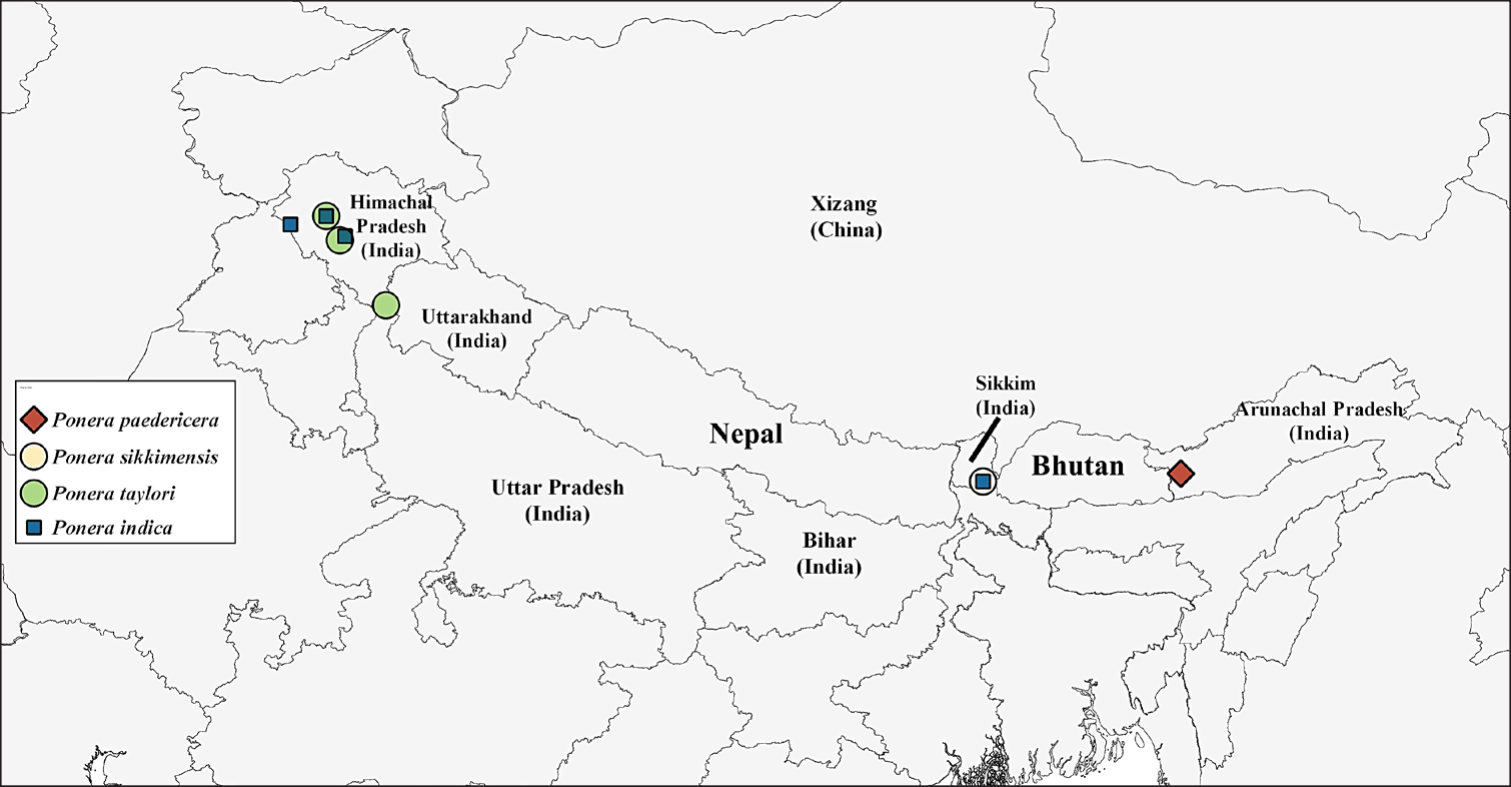
Map showing the localities from which Indian *Ponera* species have been recorded in Indian Himalaya.

#### Remarks.

The Chinese *Ponera paedericera* Zhou, 2001 is reported here for the first time from India. This species is remarkably different from the other known Indian species with following combination of characters: anterior margin of clypeus with a distinct blunt median tooth; antennal club with 3 segments; posterodorsal corners of propodeum rounded, declivity depressed, lateral sides of propodeum distinctly marginate; anterior face of petiole straight, dorsal and posterior faces form a single arched surface, anterodorsal corner blunt, dorsal surface smooth and shining, subpetiolar process with relatively large posteroventral teeth; head, mesosoma and the two basal segments of gaster densly and finely punctate; blackish in colour.

## Key to the known Indian species of *Ponera* based on worker caste

**Table:** 

1	Eyes absent; metanotal groove distinct (Fig. A); posteroventral teeth of subpetiolar process absent (Fig. C)	***Ponera taylori* Bharti & Wachkoo, 2012**
–	Eyes present; metanotal groove indistinct (Fig. B); posteroventral teeth of subpetiolar process present (Fig. D)	**2**
	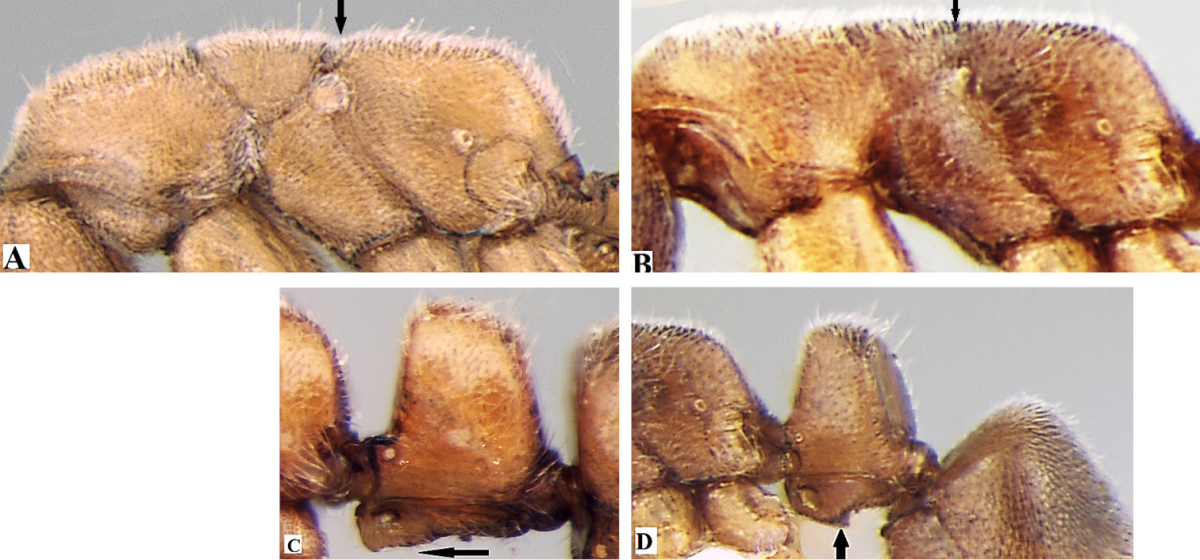	
2	A blunt tooth on clypeal margin present (Fig. E), lateral sides of propodeum distinctly marginate (Fig. G), body blackish in colour	***Ponera paedericera* Zhou, 2001**
–	A blunt tooth on clypeus margin absent (Fig. F), lateral sides of propodeum not marginate (Fig. H), body reddish brown to dark brown in colour	**3**
	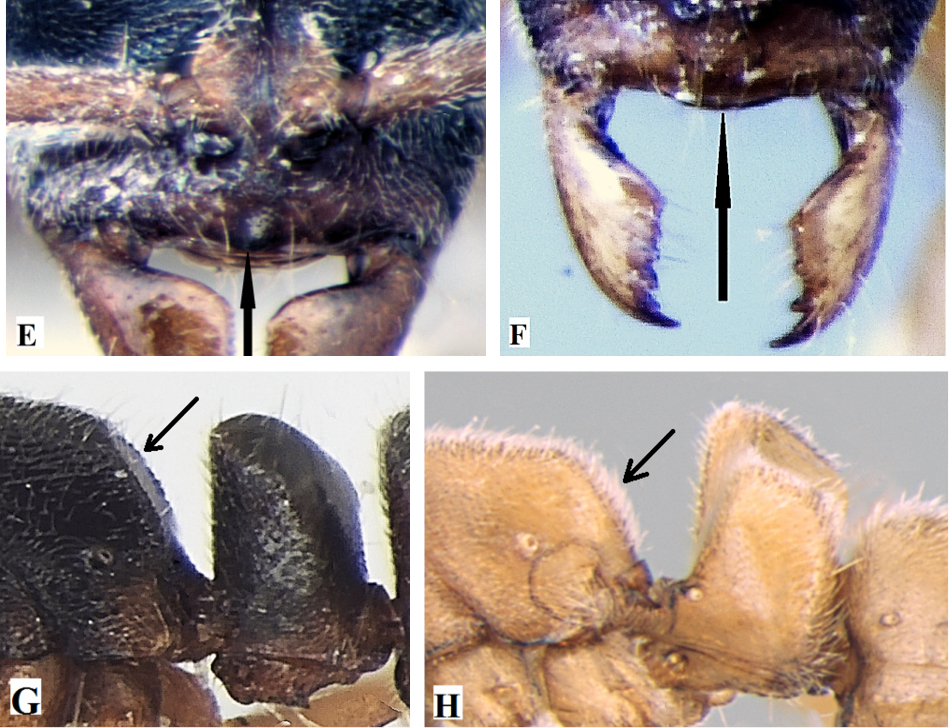	
3	Mandible with 5 well-developed teeth; mesosoma, petiole, and gaster sparsely punctate (Fig. I), teeth on subpetiolar process directed downward; (Fig. K), DPI=106	***Ponera sikkimensis* sp. n.**
–	Mandible with 3 well-developed teeth; mesosoma, petiole, and gaster densely punctate (Fig. J), teeth on subpetiolar process directed backward (Fig. L), DPI: >131–221	***Ponera indica* Bharti & Wachkoo, 2012**
	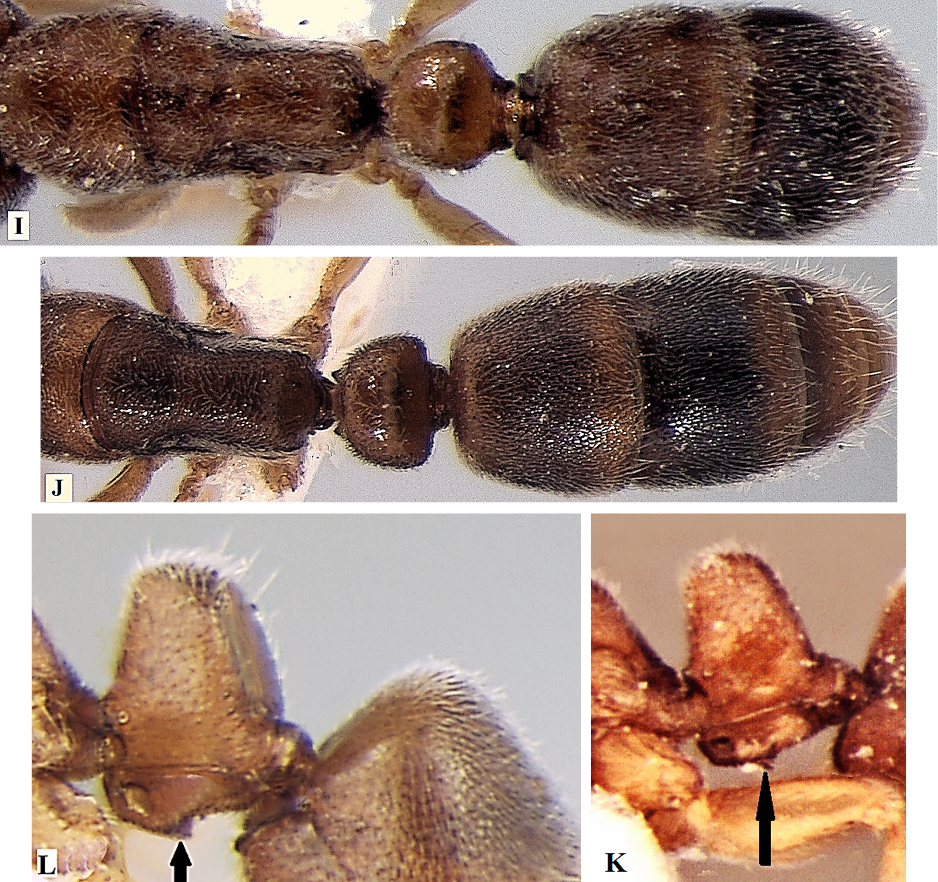	

## Supplementary Material

XML Treatment for Ponera sikkimensis

XML Treatment for Ponera indica

XML Treatment for Ponera paedericera
